# Letter to the Editor: Validity of the bolton index using cone-beam computed tomography (CBCT); methodological mistake

**DOI:** 10.4317/medoral.18642

**Published:** 2013-02-05

**Authors:** Siamak Sabour, Elahe Vahid-Dastjerdi

**Affiliations:** 1 MD, MSc, DSc, PhD Department of Clinical Epidemiology, Shahid Beheshti University of Medical Sciences, Tehran, Iran; 2 Faculty of Dentistry, Shahid Beheshti University of Medical Sciences, Tehran, Iran

## Leter to the Editor

We were interested to read the paper by Tarazona B, and colleagues published in the May 2012 issue of Med Oral Patol Oral Cir Bucal. The authors aimed to evaluate the reliability and reproducibility of calculating the Bolton Index using cone-beam computed tomography (CBCT), and to compare this with measurements obtained using the 2D Digital Method. They report by determining the regression lines for both measurement methods, as well as the difference between both of their values, the two methods are shown to be comparable, despite the fact that the measurements analysed presented statistically significant differences. (1) Why did the authors not use well known statistical tests as Sensitivity, Specificity, positive predictive value (PPV), negative predictive value (NPV) to test the validity of the Bolton Index? (2,3) or use other methods such as likelihood ratio positive and negative (LR+ & LR-)? (2,3) It is good to know that reliability (precision) and validity (accuracy) are two completely different methodological issues evaluating by different tests (2-4). As the authors point out in their conclusion; the threedimensional models obtained from the CBCT are as accurate and reproducible as the digital models obtained from the plaster study casts for calculating the Bolton Index. Such conclusion is just misinterpretation of the results and should really be avoided in clinical researches, otherwise; we will face with misdiagnosis and mismanagement of the patients.
